# Identification and Structure Analysis of *KCS* Family Genes Suggest Their Reponding to Regulate Fiber Development in Long-Staple Cotton Under Salt-Alkaline Stress

**DOI:** 10.3389/fgene.2022.812449

**Published:** 2022-02-03

**Authors:** Cun Rui, Xiugui Chen, Nan Xu, Jing Wang, Hong Zhang, Shengmei Li, Hui Huang, Yapeng Fan, Yuexin Zhang, Xuke Lu, Delong Wang, Wenwei Gao, Wuwei Ye

**Affiliations:** ^1^ Engineering Research Centre of Cotton, Ministry of Education, College of Agriculture, Xinjiang Agricultural University, Urumqi, China; ^2^ State Key Laboratory of Cotton Biology, Institute of Cotton Research of Chinese Academy of Agricultural Sciences, Zhengzhou Research Base, School of Agricultural Sciences, Zhengzhou University, Key Laboratory for Cotton Genetic Improvement, MOA, Anyang, China

**Keywords:** *Gossypium barbadense*, 3-ketoacyl-CoA synthase, long chain fatty acids, phylogenetic analysis, expression analysis

## Abstract

Plant 3-ketoacyl-CoA synthase (*KCS*) gene family catalyzed a β ketoacyl-CoA synthase, which was the rate-limiting enzyme for the synthesis of very long chain fatty acids (VLCFAs). *Gossypium barbadense* was well-known not only for high-quality fiber, which was perceived as a cultivated species of *Gossypium*. In this study, a total of 131 *KCS* genes were identified in four cotton species, there were 38, 44, 26, 23 *KCS* genes in the *G. barbadense*, the *G. hirsutum*, the *G. arboreum* and *G. raimondii*, respectively. The gene structure and expression pattern were analyzed. *GBKCS* genes were divided into six subgroups, the chromosome distribution of members of the family were mapped. The prediction of cis-acting elements of the *GBKCS* gene promoters suggested that the *GBKCS* genes may be involved in hormone signaling, defense and the stress response. Collinearity analysis on the *KCS* genes of the four cotton species were formulated. Tandem duplication played an indispensable role in the evolution of the *KCS* gene family. Specific expression analysis of 20 *GBKCS* genes indicated that *GBKCS* gene were widely expressed in the first 25 days of fiber development. Among them, *GBKCS3*, *GBKCS8*, *GBKCS20*, *GBKCS34* were expressed at a high level in the initial long-term level of the *G. barbadense* fiber. This study established a foundation to further understanding of the evolution of *KCS* genes and analyze the function of *GBKCS* genes.

## Introduction

As the basic components of lipids, fatty acids played an important role in plant growth and development and in resisting various stresses. Fatty acids can be classified according to the length of the hydrocarbon chain: short-chain fatty acids with a chain length of four to seven carbons; medium- and long chain fatty acids with a chain length of 8–18 carbons, and very long chain fatty acids (VLCFAs) with a chain length of 20 carbons and above. The biosynthesis of VLCFAs was divided into two stages and was completed in two different parts (plastids and endoplasmic reticulum) of the cell (Haslam and Kunst, 2013). The enzyme that controlled the extension of the fatty acid carbon chain was a complex that includes a variety of enzymes, including 3-ketoacyl-CoA synthase (*KCS*), β-hydroxyacyl-CoA dehydrogenase (*HCD*), β-ketoacyl-CoA reductase (*KCR*), trans-2,3-enoyl-CoA reductase (*ECR*), these four enzymes participate in the carbon chain elongation reaction and worked together to complete the synthesis of very long chain fatty acids (Kunst and Samuels, 2003; Bach and Faure, 2010). Long chain and very long chain fatty acids had a wide range of physiological functions. They participated in the formation of seed oils, biomembrane lipids and the synthesis of biologically active macromolecular sphingolipids (De Bigault Du Granrut and Cacas, 2016). In *Arabidopsis*, the hindered synthesis of VLCFAs could cause the apical meristem area of the stem to expand, and the structure of the fused rosette leaves, lateral roots, and branches changes (Faure et al., 1998; Bach et al., 2008; Beaudoin et al., 2009). VLCFAs can act as intercellular signals, inhibit cytokinin synthesis, regulate the proliferation of *Arabidopsis* stem apex cells (Nobusawa et al., 2013), and can also inhibit the totipotency of *Arabidopsis* pericylindrical cells and inhibit their formation of callus (Shang et al., 2016).

VLCFAs regulated fiber cell differentiation possibly via a lipid-activated transcriptional mechanism and may play an important role in fiber-cell initiation (Wang et al., 2015). Cotton fiber was a single-celled trichome differentiated from the epidermis of the ovule. It was a good model for studying cell elongation (Pang et al., 2010; Qin and Zhu, 2011). In cotton, in the ovule culture medium, exogenous application of saturated VLCFA can significantly promote the elongation of upland cotton fiber cells. During the development of cotton fiber, several *KCS* genes were highly upregulated, very long chain fatty acids (C20:0 to C30:0) significantly promoted cotton fiber cell elongation (Qin et al., 2007a; Qin et al., 2007b). Studies on the preferential expression of encoding fatty acid desaturases genes in fiber and exogenous application of linolenic acid (C18:3), soybean L-α-PI (PI: phosphatidylinositol), and phosphatidylinositol monophosphate (PIP) containing PIP 34:3 to promote fiber elongation had shown that fatty acids were involved in fiber elongation (Liu G.-J. et al., 2015).

*KCS* gene was responsible for the synthesis of wax component precursors and was the key rate-limiting enzyme for the extension of VLCFAs (Fan et al., 2018). Whole genome identification helps to understand and study gene function. 28, 16, 58, and 30 *KCS* genes were identified in apple (Lian et al., 2020), *Citrus sinensis* (Yang et al., 2021), *Brassica napus* (Xue et al., 2020), and *Arachis hypogaea* (Huai et al., 2020), respectively. There were 21 gene members in the *KCS* family in *Arabidopsis* (Joubès et al., 2008). Two domains: an ACP_syn_III_C domain and the Type III polyketide synthase-like protein domain had been proven necessary for KCS (Sagar et al., 2015; Guo et al., 2016; Huai et al., 2020). Researchers divided the KCS protein into four groups: FAE1, KCS1, FHD and CER6 based on the amino acid sequence homology (Costaglioli et al., 2005). The first identified gene in the *KCS* family was *KCS18/FAE1*, which could catalyze the elongation of C20 and C22 fatty acids in seeds (Rossak et al., 2001). In *Arabidopsis*, the gene *KCS* isolated from *Lunaria annua* caused a 30-fold increase in nervonic acid (*cis*-tetracos-15-*enoic* acid) proportions in seed oils (Guo et al., 2009). *Arabidopsis* and *Brassica carinata* were transformed with the *Cardamine KCS*, the C24:1 content of transgenic plants was increased 15-fold (Taylor et al., 2009). Two *KCS* genes *WSL1* and *ONI1* in rice were essential genes for leaf epidermal wax biosynthesis and the normal development of shoots (Lee and Suh, 2015).

*Gossypium barbadense* was pioneer species in the saline and alkaline land, was well-known for high-quality fiber. The completion of the splicing of the genomes of the four cotton species provides a basis for the comprehensive identification of the *KCS* genes of the four cotton species (Wang K. et al., 2012; Li et al., 2014; Hu et al., 2019). In our research, the *KCS* gene was comprehensively identified, and the evolution, chromosome location, and collinearity of the *KCS* gene were analyzed. It also focused on the analysis of the gene structure and promoter of *GBKCS* gene, and explored the expression characteristics of *GBKCS* gene during fiber development. This study established a foundation to further study of the mechanism of action of this family of genes and the biological function of *KCS* genes in the development of *G. barbadense* fibers.

## Materials and Methods

### Sequence Identification

The complete genome sequence data of four cotton species, *Gossypium barbadense* (ZJU), *Gossypium hirsutum*, (ZJU), *Gossypium arboreum* (CRI), and *Gossypium raimondii* (JGI), (Zhu et al., 2017) were obtained from Cotton FGD (Cotton Functional Genomics Database) (https://cottonfgd.org/). The amino acid sequences of *KCSs* from *Arabidopsis thaliana* were acquired from TAIR 10 (http://www.arabidopsis.org). The methods (hmmsearch and blastp) were used to identify the KCS proteins in cotton. HMMER (hmmer 3.2.1) was run based the HMM files in the local. Blastp was implemented with e-values of 1e-5 by BLAST (blast-2.12.0+). The amino acid sequences of the 3-Oxoacyl-[acyl-carrier protein (ACP)] synthase III C domain (Pfam: PF08541) and the Type III polyketide synthase-like protein domain (Pfam, PF08392) were used as queries.

### Phylogenetic Analysis and Chromosome Location

To study the evolutionary relationship between *KCS* genes of different cotton species, the protein sequence was aligned by using the ClustalW program of MEGA7.0 software. The rootless phylogenetic tree was also constructed by MEGA7.0, and the parameters were set as follows: neighboring statistics, bootstrap replication 1,000 to validating the phylogenetic tree, and intraspecific phylogenetic relationships of *KCS* genes were all constructed by the same method (Tamura et al., 2013). *G. barbadense* reference genome gff3 downloaded from Cotton FGD file and gene ID file were used to map the chromosome positions of family members through TBtools software, the position of *KCSs* in the chromosomes of *G. hirsutum*, *G. arboreum* and *G. raimondii* was mapped by the same method.

### Analysis of Protein Conserved Domains and Gene Structure

MEME (http://meme-suite.org/) website was used to identify the conserved sequence of the protein. The maximum motif parameter of the gene is 15, the other of the parameters remain unchanged, and the domain files of the *GBKCS* family genes were obtained accordingly. The protein domain was analyzed through the TBtools software.

### Analysis of the Promoter Region and Differential Expression

The 2000 bp DNA sequences upstream of *GBKCSs* gene were obtained from Cotton FGD (Cao et al., 2019; Malik et al., 2020). The cis-regulatory elements in the promoter region of the gene *GBKCSs* were predicted on the Plant CARE website (http://bioinformatics.psb.ugent.be/webtools/plantcare/html/), choosing the hormone (IAA: auxin- AuxRR-core, ABA: abscisic acid- ABRE, SA: salicylic acid, MeJA: methyl jasmonate- CGTCA-motif, TGACG-motif), transcription factor binding, development, circadian control, defense and stress responsiveness, meristem expression related cis-acting elements to analysis. We used TBtools software settng parameters (upstream bases: 2000, check retain only upstream) to check the sequence. The *GBKCSs* under different stress (salt, 100 mM NaCl, alkaline, 50 mM Na_2_CO_3_, treated for 12 h) were selected. RNA-Seq data (PRJNA734700) was downloaded from National Center for Biotechnology Information (NCBI) (https://www.ncbi.nlm.nih.gov/. nih. gov/). FPKM (fragments per kilobase of exon model per million mapped fragments) were used to analyze the expression level. Following, the summary graph which included cis-acting elements, evolutionary relationships and heatmaps was drawn by using TBtools software.

### Collinearity Analysis

*KCSs* gene locus were obtained from the gff3 file. The gff files of the genome were merged as needed. The protein sequence of all the genes were aligned, then the gene pair file of every two cotton species were obtained by using MCScanX. The collinearity analysis results were demonstrated from TBtools by combining the chromosome length files.

### Plant Materials

The ovule and fiber of cotton (*G. barbadense* cv Jiza 67) plants was used as experimental material. The flowers were tagged on the day of blooming [0 day post anthesis (DPA)] for collecting cotton fibre samples. Ovules (0 DPA) were carefully peeled off from developing cotton bolls. Fibre samples (5 DPA, 10 DPA, 15 DPA, 20 DPA and 25 DPA) were separated from developing cotton seeds. Ovules and fibre from different plants were tidied and immediately stored in a refrigerator at −80°C for further use.

### Real-Time PCR

The total RNA of the ovules and fibre were extracted separately according to the RNAprep Pure Plant Plus Kit (Polysaccharides andPolyphenolics-rich) of TIANGEN instruction. The cDNA of each sample was prepared by TransScript@ All-in-One First-Strand cDNA Synthesis SuperMix for qPCR (One-Step gDNA Removal) instructions. The primer sequences are listed in the Supplementary Table S1. The equipment used for qRT-PCR was Applied Biosystems@ 7,500 Fast, and the fluorescence quantification kit was TransStart Top Green qPCR SuperMix. The ΔΔCt method (Livak and Schmittgen, 2001) was used to calculate the processing results which contained three biological replicates.

## Results

### Identification of *KCS* Family Members

In preliminary analysis, 131 *KCS* genes were identified as *KCS* family members from four cotton species (*G. barbadense*, *G. hirsutum*, *G. arboreum*, and *G. raimondii*) (Supplementary Table S2). A total of 38 *KCS* genes were identified in *G. barbadense*. The encoded protein ranges from 411 (*GBKCS4*) to 580 (*GBKCS30*) amino acids, with pI varying from 8.281 (*GBKCS36*) to 9.732 (*GBKCS4*) and MWs varying from 46.015 (*GBKCS4*) kDa to 65.427 kDa (*GBKCS30*) (Supplementary Table S3). Through the similarity of the number of genes in two tetraploid and the sum of diploid, we found the KCS gene number of two diploid cotton species was higher than *Arabidopsis* (Joubès et al., 2008). The result indicated that the *KCS* family in cotton has undergone enlargement during the evolution. They were named *GaCXE1-26*, *GrCXE1-23*, *GBKCS1-38*, *GHCXE1-44*, according to their position on the chromosome in four different cotton variants.

### Analysis of the Evolutionary Relationship of *KCS* Gene Family Members

**FIGURE 1:**
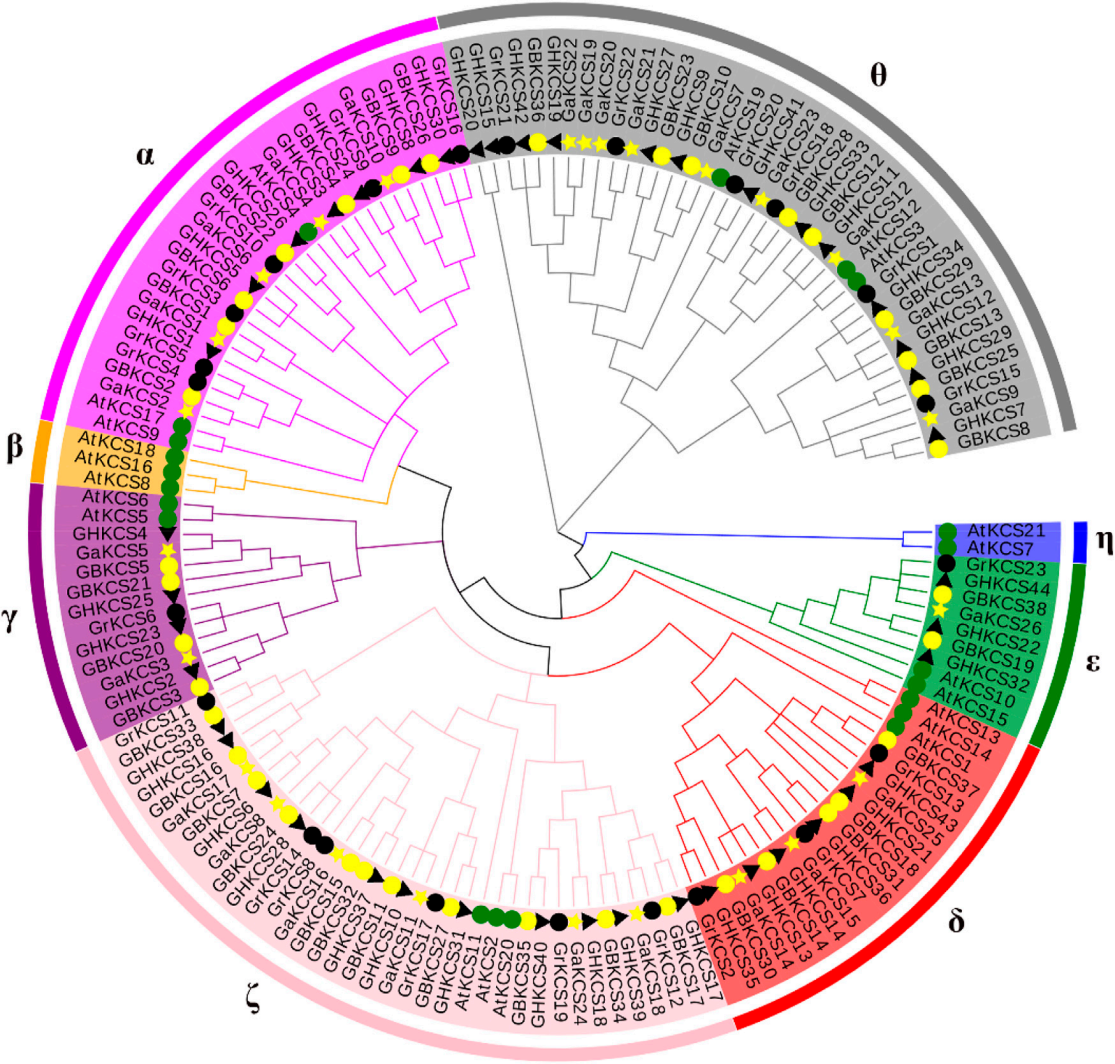
Phylogenetic analysis of KCS protein from *Gossypium* and *Arabidopsis*. (The rootless phylogenetic tree was constructed by MEGA7.0).

To clarify the evolutionary and relationship between *G. barbadense* (*GB*), *G. hirsutum* (*GH*), *G. arboreum* (*Ga*), *G. raimondii* (*Gr*), *Arabidopsis* (*At*), a total of 152 protein sequences were used for sequence alignment. The evolutionary relationship between each sequence was constructed and the rootless phylogenetic tree was generated by using MEGA7 neighbor-joining method (Figure 1). The KCS proteins were divided according to the number and structural characteristics of the domains in *Arabidopsis* (Joubès et al., 2008). All the 152 protein sequences were parted into eight subclasses: α, β, γ, δ, ε, ζ, η and θ. The results showed that, in addition to β and η, the KCS proteins of the four cotton species were distributed in each group, and the number of KCS proteins in α, ζ, δ, θ subclasses was much more than that of the other two groups.

### Analysis of Conservative Protein Motif and Gene Structure

**FIGURE 2:**
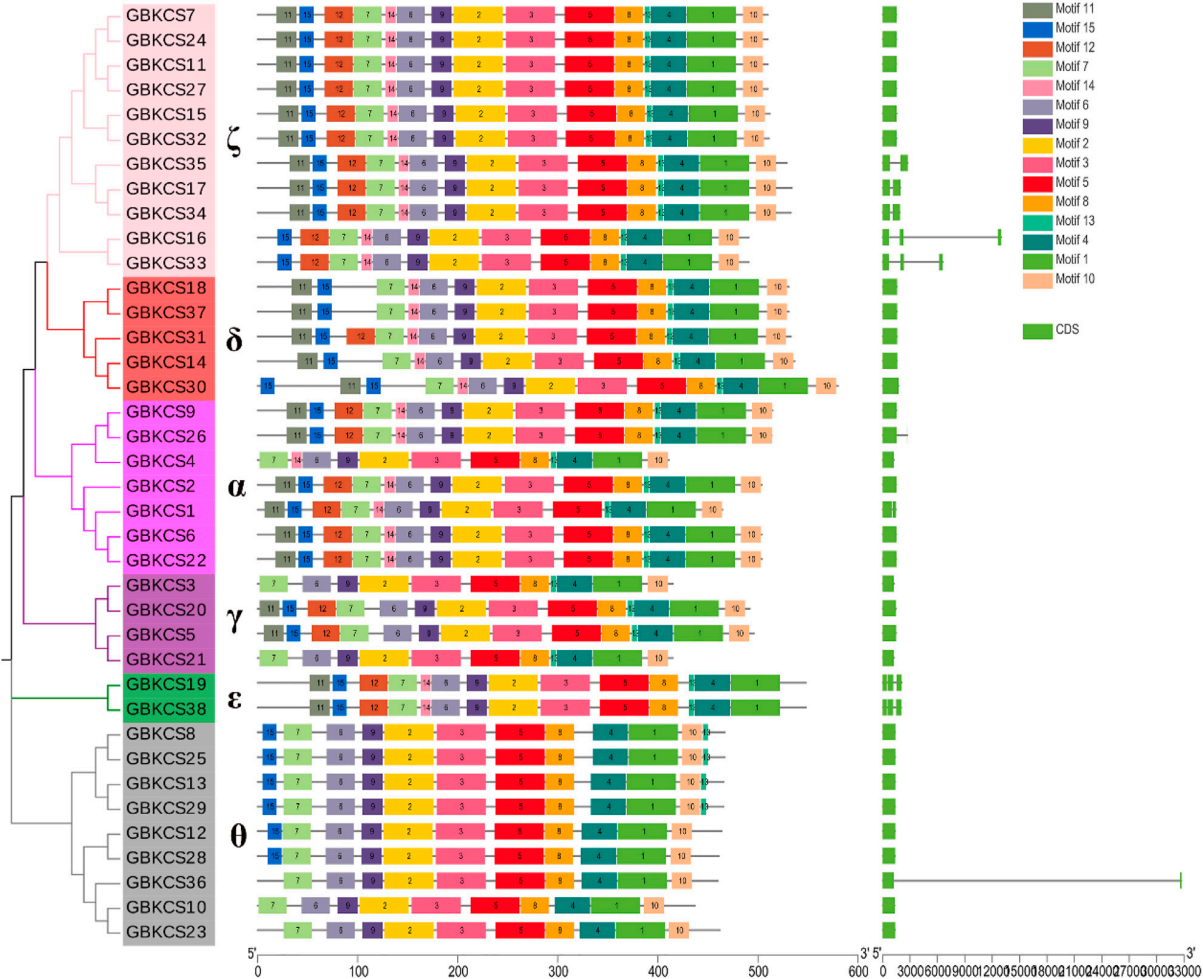
Conserved motifs and exon-intron organization of *KCS* genes from *G. barbadense*.

To explore the conservative structure of *KCS* gene family, the amino acid sequence encoded by the *G. barbadense KCS* gene was analyzed through the online website MEME. These motifs performed different functions and were distributed in the sequence of each subgroup (Figure 2). Compared with other subgroups, all members (*GBKCS19*, *GBKCS38*) of the ε subgroup did not contain motif 10, and consisted of three adjacent exons. In the θ subgroup, three members (*GBKCS23*, *GBKCS10*, *GBKCS36*) lacked motif 15, we also noticed that the *GBKCS23*, *GBKCS10*, *GBKCS36*, *GBKCS12*, and *GBKCS28* of the subgroup did not contain motif 13. In the γ subgroup, the sequences of *GBKCS3* and *GBKCS21*, *GBKCS20* and *GBKCS5* were highly similar and consisted of the same motifs respectively. *GBKCS4*, a member of the α group, lacked motif 15, and *GBKCS1* had two exons. The similar motif arrangements in the same subgroup implicited that the protein structure was conserved in a specific subgroup, the gene had a certain degree of conservation, and the functions of most conserved motifs remain to be elucidated.

### Chromosomal Localization of *KCSs* in Four Gossypium Species

**FIGURE 3:**
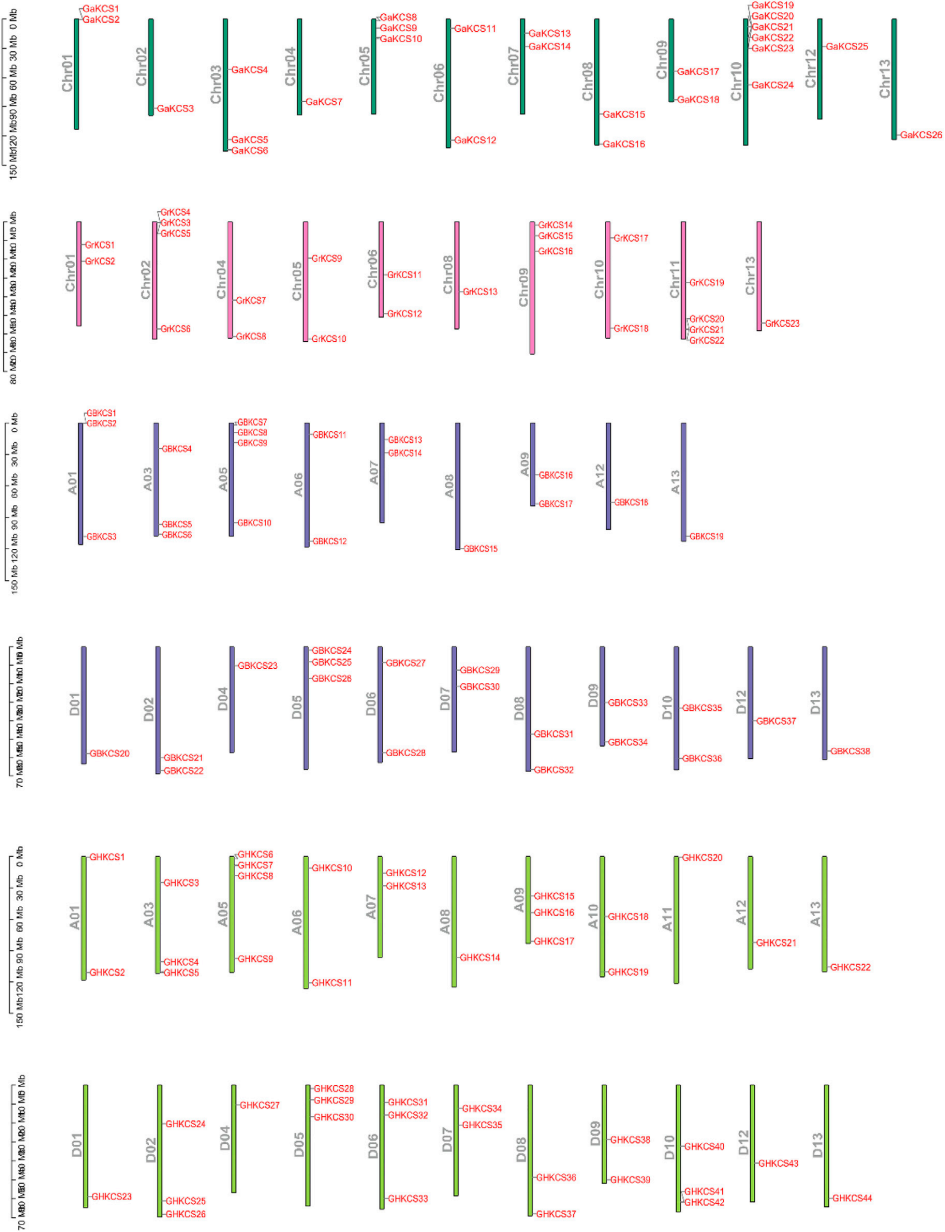
Chromosomal location of *KCS* genes in four cotton species.

**FIGURE 4:**
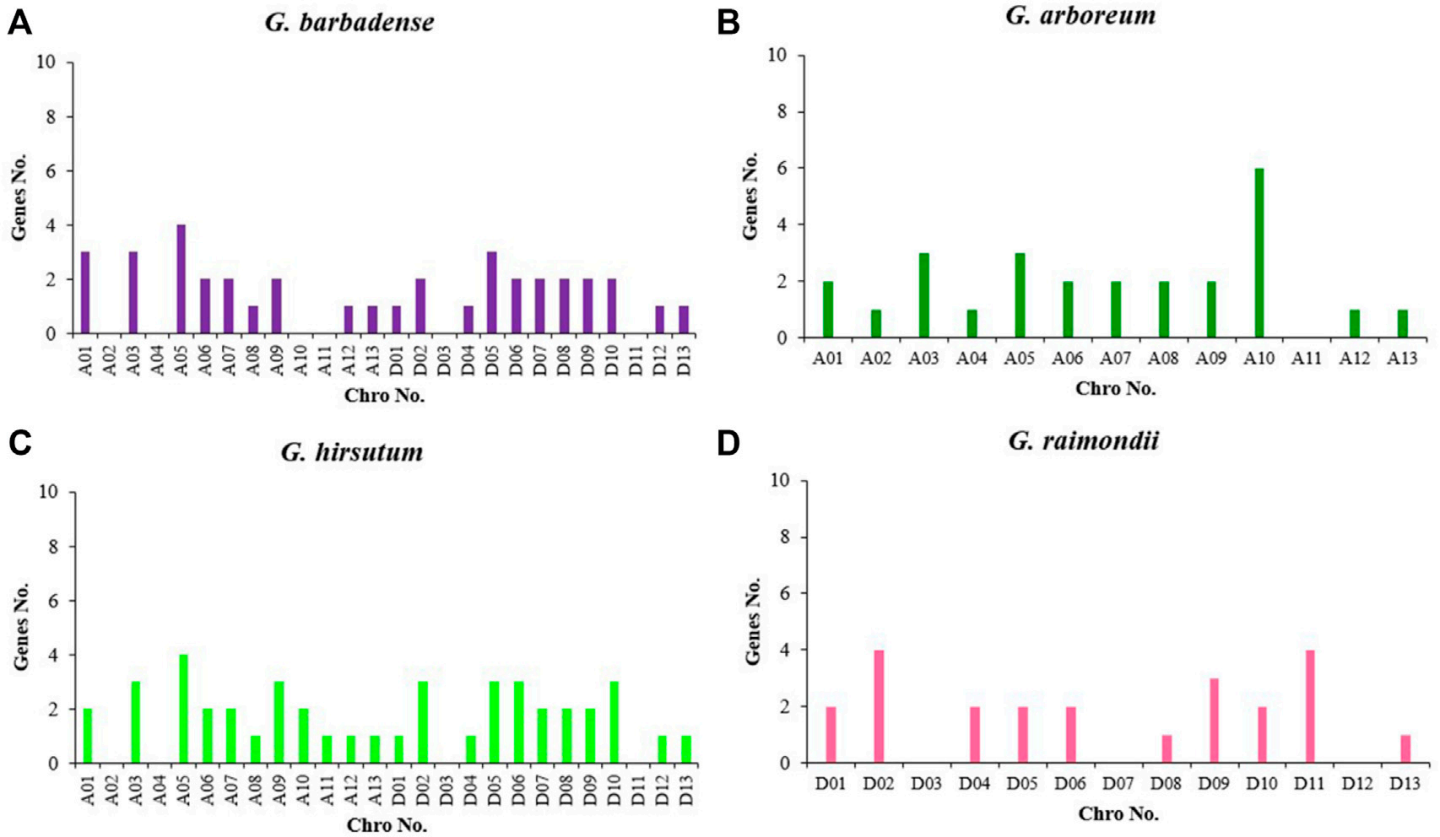
Distribution of the number of genes in the *KCS* genes family of four cotton species. **(A)** *G. barbadense*; **(B)** *G. arboreum*; **(C)** *G. hirsutum*; **(D)** *G. raimondii*.

The gff3 file of the genome and the gene ID information were used to understand the specific distribution of genes on chromosomes more intuitively. The *KCS* genes of four cottons were located on the chromosomes of the corresponding cotton species (Figure 3). The *KCS* genes of *G. arboreum* were located on 12 other chromosomes except chromosome 11 (Figure 4B), among them, the number of genes on chromosome 10 was the largest, with a total of six genes (*GBKCS19-24*). In *G. raimondii*, D03, D07, D12 of this cotton species had no *KCS* family members (Figure 4D). The number of genes on the chromosomes of the two subgroups was the same in two tetraploid, and the genes are located in the same D_1_t and D_2_t chromosomes. The distribution of the *KCS* gene on the A subgenome chromosomes of *G. barbadense* (AD_2_) and *G. hirsutum* (AD_1_) was quite different (Figures 4A,C), that may because the addition or loss of genes through duplication events (entire genome duplication, tandem duplication, fragment duplication).

### Analysis of Promoters and Expression of *GBKCS* Gene Family Members Under Salt-Alkaline Stresses

**FIGURE 5:**
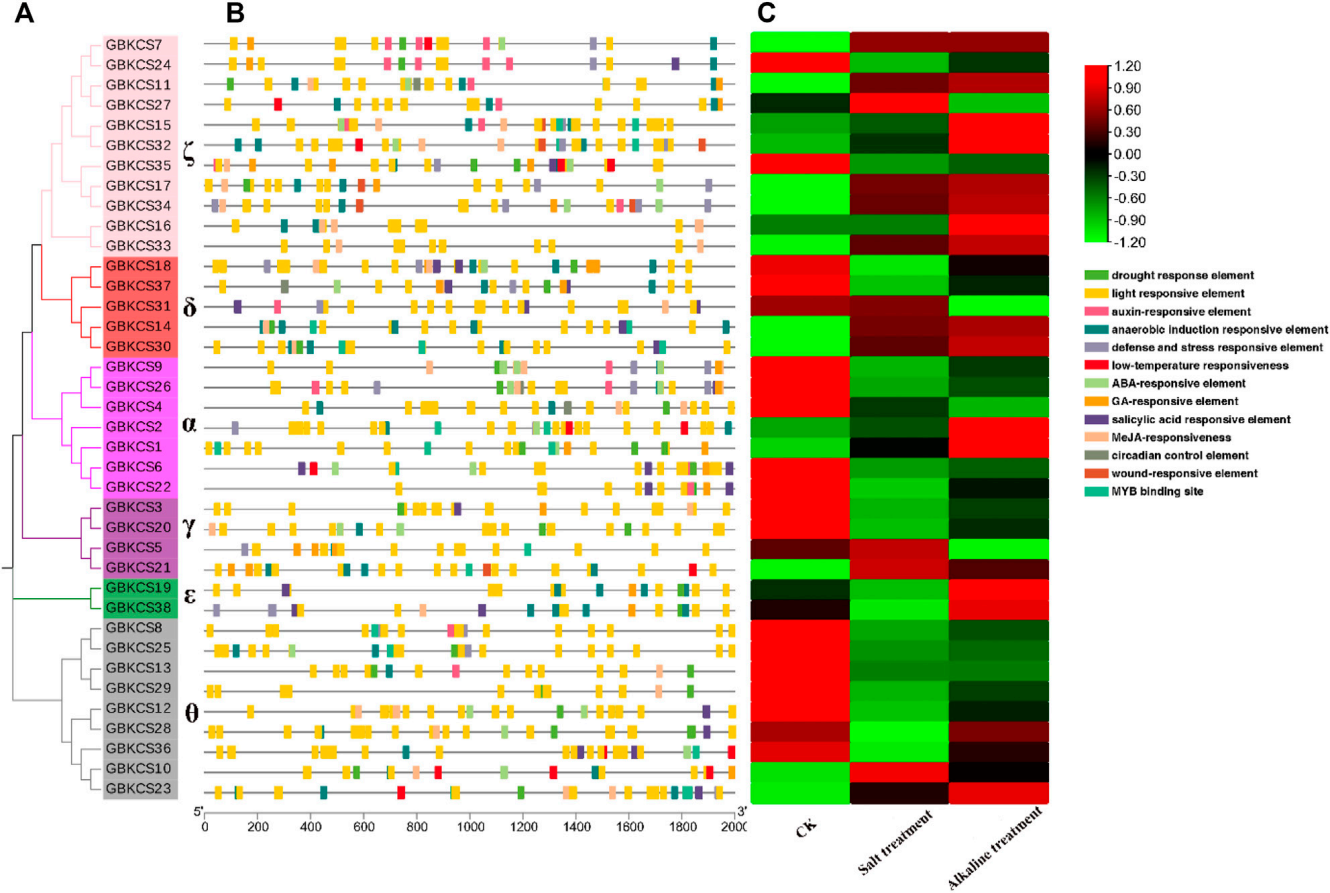
Analysis of promoters and differentially expressed genes of *GBKCSs* family. **(A)** Phylogenetic tree of *GBKCSs*; **(B)** Cis-elements in promoters of *GBKCS* genes; **(C)** Differentially expressed genes of *GBKCS* genes under salt and alkaline stress. (The control sample was designated as CK. The Na_2_CO_3_ processed sample designated as Alkaline, and the NaCl processed sample designated as Salt).

Cis-acting elements occur in the promoter region upstream of the gene, which may bind to transcription factors for regulating gene transcription and responding to different environmental factors. The response element of *GBKCSs* were identified by using the evolutionary relationship and the 2 kb region file upstream of the start codon. These cis-acting elements were involved in the hormone response, stress induction, and the defense reaction, implying that KCS protein played important roles in hormone response and stress induction in plants. Most of the promoters from *GBKCS* gene family members contain drought, light, low-temperature, ABA, GA, MeJA, MYB response elements (Figure 5B) (Supplementary Table S5). We can explore and understand the mechanism of gene response to different plant hormones and abiotic stress through promoter analysis. To further investigate the responsive mechanism of *GBKCS* gene family members against abiotic stresses, RNA-Seq data was used to determine the differences in gene expression of family members under salt-alkaline stress (Figure 5C). Under the salt-alkali stress, the expression level of all members has changed to a certain degree. Except for *GBKCS10* and *GBKCS23*, the expression of other genes in the θ subgroup (Figure 5A) showed a downward trend after salt-alkali stress. Two genes in the ε subgroup (*GBKCS19*, *GBKCS38*) showed high expression levels. Interestingly, gene expression changed under different stresses, even though in the same subgroup, the functions of gene members were also diverse. These results indicated that *GBKCS* members were involved in the regulation of abiotic stress, the response expression of different members was different, and the expression of the same gene under different stress was also different.

### Duplication and Collinearity Analysis of *KCS* Gene

**FIGURE 6:**
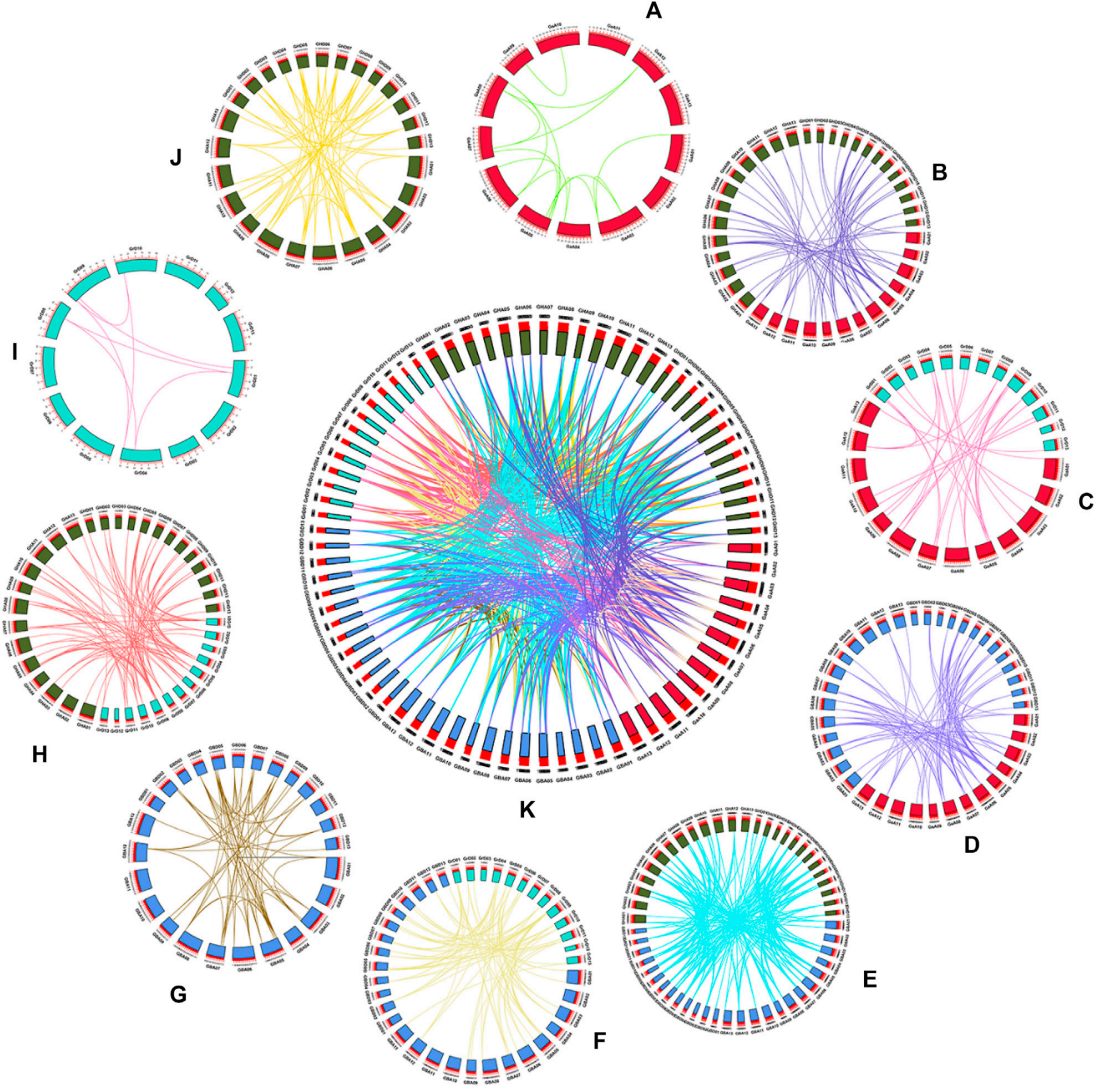
The collinearity of *KCS* genes within and among the four cotton species genomes (*Gossypium hirsutum*, *Gossypium barbadense*, *Gossypium arboreum* and *Gossypium raimondii*). Chromosomal lines represented by various colors indicate the syntenic regions around the *KCS* genes. **(A)** Ga-Ga, **(B)** Ga-GH, **(C)** Ga-Gr, **(D)** Ga-GB, **(E)** GB-GH, **(F)** GB-Gr, **(G)** GB-GB, **(H)** Gr-GH, **(I)** Gr-Gr, **(J)** GH-GH, **(K)** Summary of 10 combinations of collinearity.

Whole genome duplication, segmental duplication and tandem duplication were three processes of gene families arises evolution (Xu et al., 2012). To describe the positional relationship of the homology of genes, the amplification mechanism and the sequence of arrangement, we performed a collinearity analysis on the *KCS* genes of the four cotton species (Figure 6). MCScanX (Wang Y. et al., 2012) was used to find homologous gene pairs, a total of 680 gene pairs were identified. 2 (*GaKCS19*- *GaKCS20*, *GaKCS20*- *GaKCS21*) and 1 (*GrKCS4*- *GrKCS5*) pair of tandem repeats were identified in Ga-Ga and Gr-Gr, respectively. The two Ga-Ga gene pairs were in the same branch of the evolutionary tree, and the Gr-Gr tandem repeat gene pair was a homologous gene pair. There was a pair of tandem repeats in GB-GB, while no pair was identified in GH-GH. Tandem duplication played an indispensable role in the evolution of the *KCS* gene family. 15, 7, 66, 59 gene pairs were identified in Ga-Ga, Gr-Gr, GH-GH, GB-GB, respectively. The Gr and Gr collinearity gene pairs were the least among the groups, with seven pairs, meanwhile GB and GH was the most, with 172 pairs. From the perspective of the number of gene pairs, this was consistent with the number of *KCS* genes in diploid and tetraploid genes.

### Selective Pressure Ka/Ks Measurement and Analysis

**FIGURE 7:**
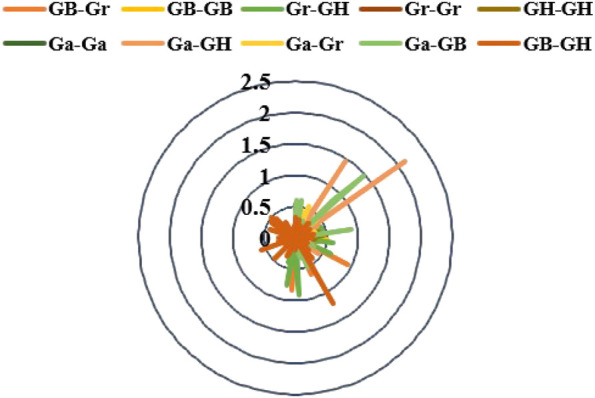
Select the radar chart for pressure (Ka/Ks) analysis.

Gene duplication may contribute to the expansion of gene families. The selection pressure of gene duplication gene pairs in the evolution process can be studied by calculating non-synonymous (Ka) to synonymous (Ks) substitution rate. Ka/Ks > 1 and Ka/Ks < 1 were considered positive selection and purification selection, respectively (Hurst, 2002). The homologous gene pairs Ka and Ks and Ka/Ks of four cotton species in 10 combinations (Ga-Ga, Ga-GH, Ga-Gr, Ga-GB, GB-GH, GB-Gr, GB-GB, Gr-GH, Gr-Gr, GH-GH) were measured (Figure 7). In our results, there were four gene pairs with Ka/Ks > 1, and the ratio of 567 gene pairs was less than one (Supplementary Table S4), which meant that the *KCS* gene family in the four cotton species underwent strong purification selection and remained highly conserved.

### Expression Analysis of *GBKCS* Genes in Different Stages of Fiber Development

**FIGURE 8:**
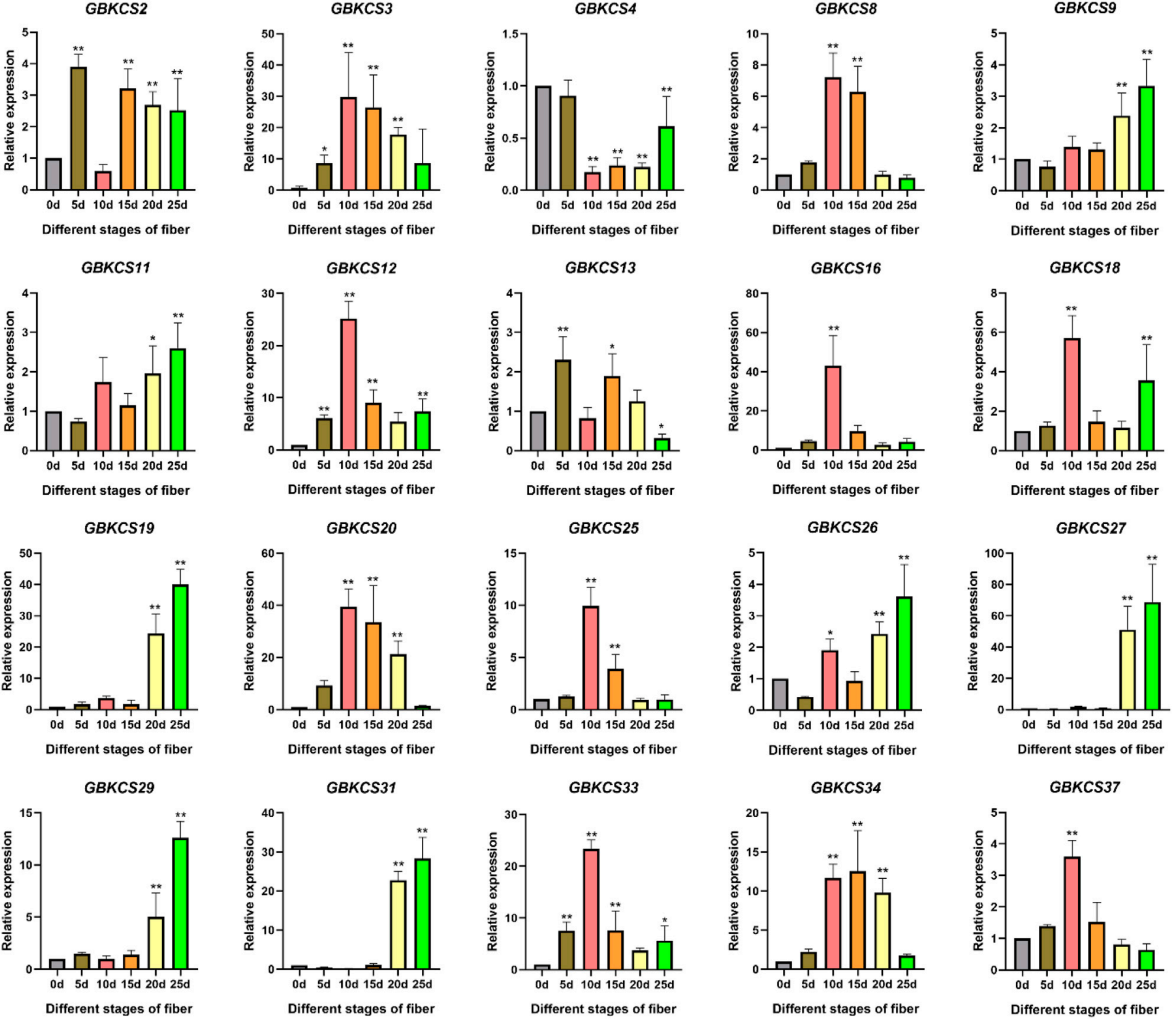
Analysis of differentially expressed *KCS* gene family members in *G. barbadense* (*p < 0.05, **p < 0.01).

**FIGURE 9:**
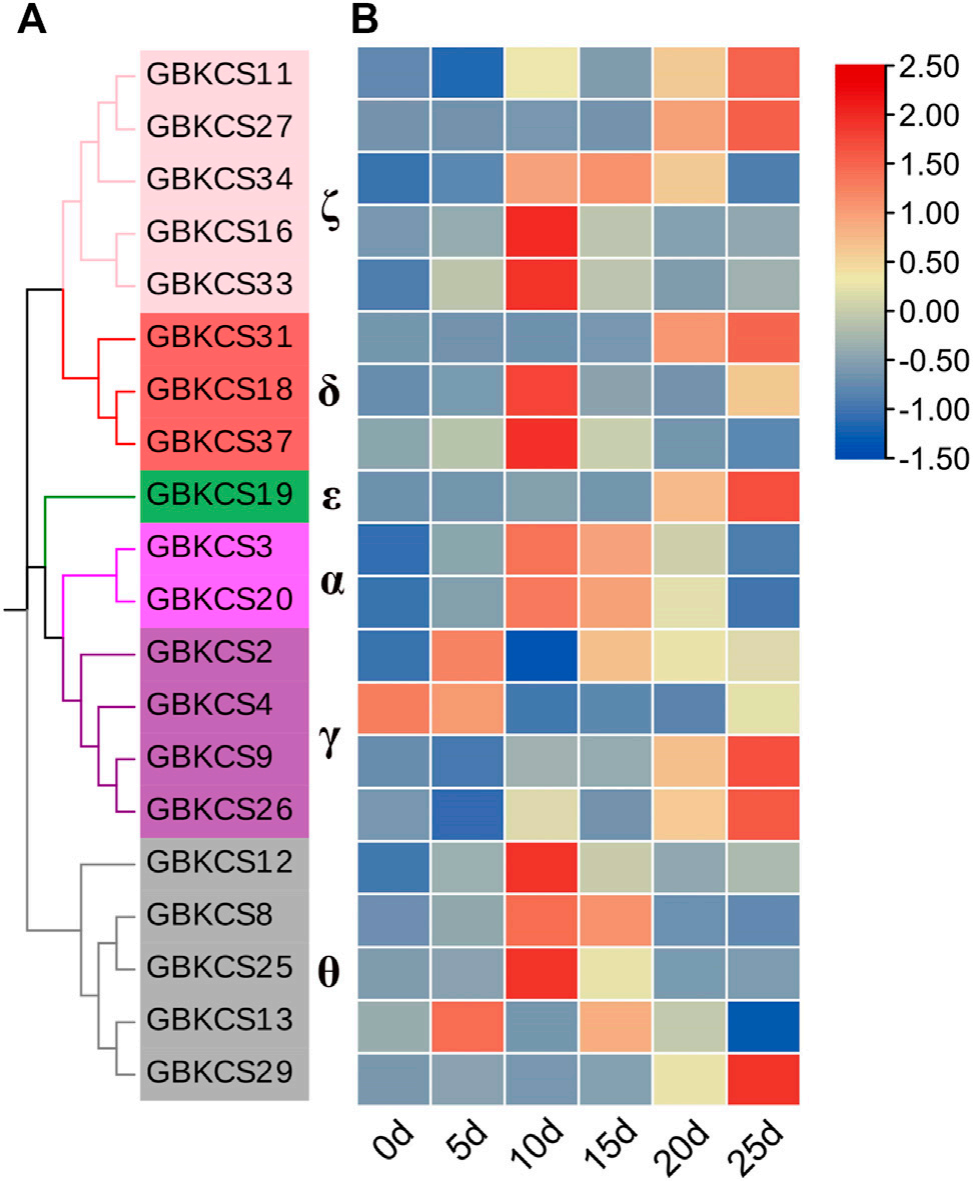
Analysis of the expression patterns of 20 genes in the *GBKCS* gene family. **(A)** The evolutionary relationship of 20 *GBKCS* genes. **(B)** Expression model of 20 *GBKCS* genes at different stages of fiber development. Each column represented the expression of different genes in the same sample, and each row represented the expression of the same gene in different samples. The heat map was drawn with TBtools software.

Research on KCS wax synthesis genes was mainly focused on oil crops. From the previous evolutionary relationship, motif and promoter element analysis of our research, we found that genes in the same subgroup have the same or similar structure and conservative motifs. In order to explore the potential role of *GBKCS* gene in the process of fiber development, ovules or fibers of different stages were prepared. According to the evolutionary relationship, a total of 20 genes were selected from each group (Figure 9A). The expression levels of selected genes at different stages of fiber development were finally determined (Figure 8). These genes were α (*GBKCS2*, *GBKCS4*, *GBKCS9*, *GBKCS26*), γ (*GBKCS3*, *GBKCS20*), δ (*GBKCS18*, *GBKCS31*, *GBKCS37*), ε (*GBKCS19*), ζ (*GBKCS11*, *GBKCS16*, *GBKCS27*, *GBKCS33*, *GBKCS34*), θ (*GBKCS8*, *GBKCS12*, *GBKCS13*, *GBKCS25*, *GBKCS29*) members of subgroups. In order to show more intuitively the expression patterns of these 20 *GBKCS* genes at different stages of fiber development, we made a heat map (Figure 9B). The representative genes of the α subgroup showed high expression levels on the 10–20 days, while the expression levels of *GBKCS2*, *GBKCS13* and *GBKCS4* were higher at the early stage of fiber development (5 days), and we found that the expression level of *GBKCS4* during 10–25 days was lower than that of the ovule. The expression levels of *GBKCS3* and *GBKCS20* genes from the same subgroup at 10, 15, and 20 days were significantly higher than that of the ovule. The expression levels of genes *GBKCS18*, *GBKCS31* and *GBKCS37* were different in different periods. The gene *GBKCS16* of the ζ subgroup was only expressed at a high level on the 10 days. The members of the θ group *GBKCS8*, *GBKCS12* and *GBKCS25* all expressed high levels on the 10 days.

### Interaction Network of GBKCS Proteins

**FIGURE 10:**
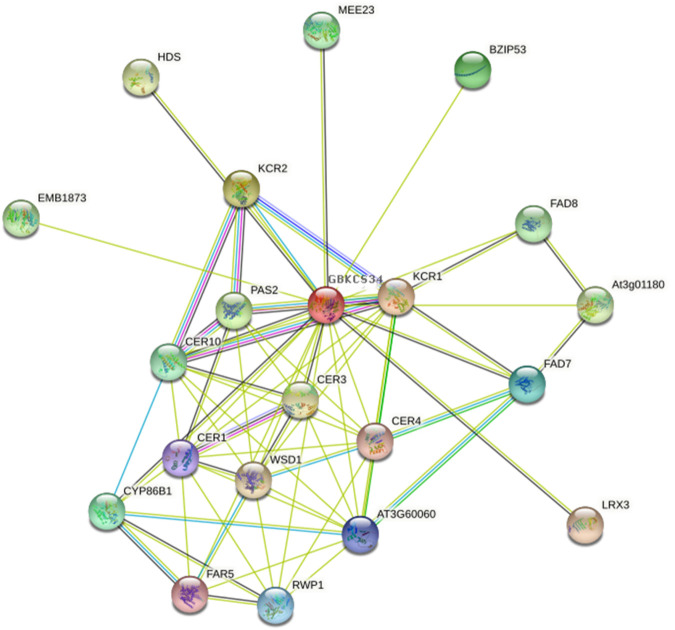
Interaction network of GBKCS proteins.

For functional analysis of GBKCS proteins, we combined the promoter element and the heat map analysis of saline-alkali stress (Figures 5B,C), and finally *GBKCS34* that was expressed at a high level after stress was selected. The homologous gene of *GBKCS34* in *Arabidopsis* was obtained according to blastp. STRING database (https://string-db.org/) was used to construct an interaction network to analyze GBKCS protein function (Figure 10). Overview of GBKCS proteins, more than 90% of the protein were involved in the pathway module of fatty acid biosynthesis, elongation, endoplasmic reticulum (M00415). The proteins were enriched in fatty acid elongation (ko00062) and biosynthesis of secondary metabolites (ko01110) metabolic pathways. According to *GBKCS34* promoter analysis, the sequence contained light, low-temperature, GA, ABA, auxin, MeJA responsive element. Take this, the function of GBKCS protein can be further deduced. The function of GBKCS protein was speculated to be based on the study of *AtKCSs*. The *Arabidopsis thaliana* ECERIFERUM1 (CER1) protein was an essential element of wax alkane synthesis, *GBKCS34* interacted with protein 3-ketoacyl-CoA reductase (*KCR1*, *KCR2*), *CER1*, *CER3*, *CER4*, *CER10*. Studies have reported that *AtCER1* and *AtCER3* were overexpressed in yeast, and it was found that *AtCER1* and *AtCER3* interacted to synthesize VLCFAs into alkanes (Bernard et al., 2012). The analysis of the KCS protein promoter elements and protein network interactions would help us understand the protein’s mechanism of action.

## Discussion

*KCS* gene exists in almost all plant species (Guo et al., 2016). The relationship between individual or group genetic differences and gene redundancy were clarified through research on gene families (Liu H.-J. et al., 2015). Our results showed that 26, 23, 38, 44 *KCS* genes had been identified in four cotton species (*G. arboreum*, *G. raimondii*, *G. barbadense*, *G. hirsutum*) (Supplementary Table S2). The number of genes in each species explained the origin of cotton tetraploid to a certain extent. Genes were often acquired and lost in the process of evolution. This increase and deletion of genes leaded to differences in the number of *KCS* genes in cotton. Based on the evolutionary relationship of the *KCS* family in *Arabidopsis* (Joubès et al., 2008), we combined the amino acid sequence to divide the *KCS* genes in the four cotton species into eight subgroups (α, β, γ, δ, ε, ζ, η and θ) (Figure 1). The analysis results of motif and gene structure (Figure 2) showed that most of *GBKCS* members were relatively conservative with one exon and no intron structure.

Replication events were one of the main drivers of evolutionary diversification of genomes and genetic systems (Gu et al., 2003). Non-functionalization, sub-functionalization and new functionalization were the three evolutionary directions of proteins (Yang et al., 2017). The total number of *Brassica napus KCS* genes was less than the sum of *Brassica rapa* and *Brassica oleracea* (Xue et al., 2020), and the members of the *Brassica napus FAD* gene family were also accompanied by a decrease in the number of genes after polyploidization (Xue et al., 2018; Xu et al., 2019). Environmental conditions and artificial selection affected the number of gene family members. The doubling of the gene sequence in cotton would occur with the occurrence of repetitive events, and some redundant genes would be selectively lost or recombined (Li et al., 2015). In our research results, consistent with *Brassica napus* was that the *KCS* gene in *G. barbadense*, *G. hirsutum* cotton was less than the sum of *G. arboreum*, and *G. raimondii*. The function of normal *KCS* genes may be affected by non-synonymous mutations, and even affected the normal growth of plants. Studies have reported that most of the *Citrinae KCS* genes were synonymous mutations (Yang et al., 2021). However, *WSL1*, a member of the rice *KCS* gene family, which was involved in leaf wax accumulation, the total amount of epidermal wax in its mutant leaves and leaf sheaths was significantly reduced, and VLCFA precursors of C20-C24 decreased in both (Yu et al., 2008). In tomato slcer6 mutants, *SlCER6* loss of function provokes a decrease of n- and iso-alkanes with chain lengths of C27 or greater and of anteiso-alkanes with chain lengths of C28 or greater in flower cuticular waxes. This mutation ultimately resulted in impaired fertility and changes in flower shape (Smirnova et al., 2013). Allotetraploid genomes had lower selection pressure than diploid genomes (Li et al., 2015). Existing studies had revealed that purification selection was the main evolutionary force acting on the *FAE1* gene (Sun et al., 2013). Consistent with this, our results showed that the Ka/Ks of more than 90% of the *KCS* gene pairs in the four cotton species were far below one. Therefore, it can be inferred that *KCS* had undergone purification selection during the evolution of cotton.

VLCFAs were the components of plant cuticle wax layer and cell plasma membrane, which played an important role in the survival and development of plants (Bernard and Joubès, 2013; Xue et al., 2017). *KCS* genes determined whether fatty acids will be extended and the number and types of VLCFAs produced (Lechelt-Kunze et al., 2003; Dongxin et al., 2015; Ozseyhan et al., 2018). The transcription of *CsKCS6* was changed in response to drought stress, salt stress and abscisic acid (ABA) treatment. *CsKCS6* was essential in the synthesis of fatty acid precursors involved in wax synthesis, and it also enhanced the tolerance of transgenic *Arabidopsis* to drought and salt stress (Guo et al., 2020). In our results, some *KCS* genes of *G. barbadense* responded to salt-alkaline stress (Figure 5C). Genes such as *GBKCS34*, *GBKCS2*, *GBKCS1*, and *GBKCS13* were significantly up-regulated, indicating that the members of the *KCS* gene family of *G. barbadense* play a role in saline-alkali abiotic stress.

To have a more comprehensive understanding of the expression patterns of *KCS* family genes, *GBKCS* genes were selected to be classified according to the subgroups of evolutionary relationships. The 20 genes we selected from each subgroup were widely expressed in the 0–25 days of fiber development (Figure 8). *GBKCS34* was a homologous gene of *AtKCS20*, all of which belonged to the ζ subgroup. *AtKCS20*, *AtKCS2*, *AtKCS5* were found to be enzymatically active with endogenous yeast fatty acid substrates and to some extent with externally supplied unsaturated substrates, involved in the synthesis of saturated and monounsaturated fatty acids 20–30 carbon atoms in length (Trenkamp et al., 2004; Paul et al., 2006). The abundance of regulatory elements in the promoter region may be the result of selective pressure (Xiao et al., 2016). Analysis of promoter action elements suggested that pathways such as low temperature induction, light, and plant hormones may regulate the transcription of *GBKCS34* by combining these action elements, and then played a regulatory role in these pathways. In addition, the expression of *GBKCS34* in 10 DPA, 15 DPA, and 20 DPA was significantly higher than the other three periods, which also indicated that this gene was involved in the development of fiber elongation.

## Conclusion

The *KCS* family genes of the *G. barbadense*, *G. hirsutum*, *G. arboreum*, *G. raimondii* genomes were analyzed. A total of 131 *KCS* genes were identified in four cotton species through screening *in silico*. We analyzed the developmental and evolutionary relationship of the four *KCS* genes, chromosome location, and collinearity. The gene structure, motif, promoter elements, and expression patterns of *G. barbadense KCS* were analyzed. The family had undergone purification selection during evolution. Some *GBKCS* genes responded to salt-alkali stress and were also widely expressed at different stages of fiber development. Furthermore, the high expression of *GBKCS3*, *GBKCS8*, *GBKCS20* and *GBKCS34* in a specific period implied the potential function of these genes in the fiber elongation period. This work had enriched our knowledge of the *KCS* gene of *G. barbadense* and laid the foundation for further exploring the mechanism of the *KCS* family gene in the development of *G. barbadense* fiber.

## Data Availability

The original contributions presented in the study are included in the article/Supplementary Material, further inquiries can be directed to the corresponding authors.

## Supplementary Material

Supplementary Table S1 Primer sequence of *GBKCS*.

Supplementary Table S2
*KCS* members of the four cotton species.

Supplementary Table S3 Physical properties of GBKCS proteins.

Supplementary Table S4 Prediction of duplicated gene pairs involved in different combinations from four *Gossypium* species.

Supplementary Table S5 Promoter sequence and classification information.
